# Liver proteomic analysis reveals the key proteins involved in host immune response to sepsis

**DOI:** 10.7717/peerj.15294

**Published:** 2023-05-26

**Authors:** Yingying Chen, Hui Gong, Donge Tang, Lan Yu, Shoubin Long, Bao Zheng, Dixian Luo, Anji Cai

**Affiliations:** 1 Huazhong University of Science and Technology Union Shenzhen Hospital, Shenzhen, China; 2 Clinical Medical Research Center, The Second Clinical Medical College of Jinan University, Shenzhen People’s Hospital, Shenzhen, China

**Keywords:** Proteomic analysis, Rat model, Sepsis, TMT, RPM

## Abstract

**Background:**

Sepsis is a serious infection-induced response in the host, which can result in life-threatening organ dysfunction. It is of great importance to unravel the relationship between sepsis and host immune response and its mechanisms of action. Liver is one of the most vulnerable organs in sepsis, however, the specific pathogenesis of septic liver injury has not been well understood at the protein level.

**Methods:**

A total of 12 healthy Sprague–Dawley (SD) male rats aged from 6 to 8 weeks were adaptively housed in individual cages in the specific pathogen free animal room. These lab rats were grouped into two groups: treatment (*N* = 9) and control (*N* = 3) groups; only three mice from the treatment group survived and were used for subsequent experiments. A TMT-based proteomic analysis for liver tissue was performed in the septic rat model.

**Results:**

A total of 37,012 unique peptides were identified, and then 6,166 proteins were determined, among which 5,701 were quantifiable. Compared to the healthy control group, the septic rat group exhibited 162 upregulated and 103 downregulated differentially expressed proteins (DEPs). The upregulated and downregulated DEPs were the most significantly enriched into the complement and coagulation cascades and metabolic pathways. Protein-protein interaction (PPI) analysis further revealed that the upregulated and downregulated DEPs each clustered in a PPI network. Several highly connected upregulated and downregulated DEPs were also enriched into the complement and coagulation cascades pathways and metabolic pathways, respectively. The parallel reaction monitoring (PRM) results of the selected DEPs were consistent with the results of the TMT analysis, supporting the proteomic data.

**Conclusion:**

Our findings highlight the roles of complement and coagulation cascades and metabolic pathways that may play vital roles in the host immune response. The DEPs may serve as clinically potential treatment targets for septic liver injury.

## Introduction

Sepsis is a serious infection-induced response in the host and is a major cause of morbidity and mortality worldwide (Salomão et al., 2019). Additionally, the disease has a high morbidity rate, rapid disease progression, and a poor prognosis. Sepsis has been the subject of basic and clinical research for both domestic and foreign scholars, but clinical translation results are limited, and the incidence of this disorder continues to rise. Therefore, as a systemic inflammatory response caused by infection, sepsis will benefit from immunoadjuvant therapy, which is expected to make significant strides in the treatment of the disease.

Evidence showed that both inflammation and the immune system play major roles in the development of sepsis, and immune dysfunction is key to the development of the condition (Nedeva, Menassa & Puthalakath, 2019). Studies indicate that sepsis can be caused by multiple molecular mechanisms, including a dysfunctional innate and adaptive immune system and multiple downstream events (Bosmann & Ward, 2013; Minasyan, 2019; Nedeva, Menassa & Puthalakath, 2019). The cytokines play a pleiotropic role in the regulation of both innate and adaptive immune systems (Oppenheim, 2001). Multiple prototype inflammatory cytokines including tumor necrosis factor (TNF-a), interleukins (IL-1b and IL-6), monocytic chemotactic protein (MCP-1), nuclear transcription factor (NFκB), Toll-like receptor 4 (TLR-4) were successively identified and used for unveiling immunopathological processes in sepsis (Ono et al., 2018; Hunt, 2019). Moreover, oxidative stress (Mantzarlis, Tsolaki & Zakynthinos, 2017), microcirculation dysfunction (Charlton et al., 2017), Mitochondrial fusion and disintegration (Arulkumaran et al., 2016), and apoptosis (Mahidhara & Billiar, 2000) have been demonstrated to be associated with molecular mechanisms of organ damage in sepsis. In addition, there is growing evidence that miRNAs regulate a variety of metabolic pathways including innate immune processes, apoptosis, and mitochondrial function that may play a role in the pathophysiology of sepsis (Wang et al., 2012; Yang et al., 2021; Benz et al., 2016). In particular, there is strong evidence that the TLR4/NFκB signaling contributes to the pathogenesis of sepsis by increasing the production of inflammatory mediators (Kuzmich et al., 2017). However, attempts to improve sepsis therapeutic outcomes by targeting proinflammatory mediators, such as TNF and IL1 antagonists and TLR blockers, have been unsuccessful. Therefore, unraveling the relationship between sepsis and host immune response and its mechanisms of action will require continued research.

As the body’s largest gland, the liver is crucial to metabolic and immunological balance. In previous studies, it has been demonstrated that the liver is a double-edged sword in sepsis as a lymphoid organ responding to sepsis; liver-mediated immune responses can clear bacteria and toxins, but they can also result in inflammation, immunosuppression, and organ damage (Yan, Li & Li, 2014). Meanwhile, exploring the inflammatory pathogenesis of sepsis in liver has become one of the concerns of the scientific community. Wang et al. (2019) demonstrated that some proinflammatory factors were detected in exosomes released from LPS-induced macrophages, which contribute to sepsis-induced acute liver injury by regulating multiple inflammatory pathways. Chen et al. (2007) highlighted that the ALDH2 might play a functional role in the pathogenesis of sepsis and provide a novel protective mechanism of heat shock treatment using the liver mitochondrial proteins analysis. Sehgal et al. (2022) have demonstrated that several autophagy proteins including DNAJC13, AHSG, TMSB4X, PROS1 and SERPINA3, which can be used as therapeutic targets in decompensated cirrhosis patients with sepsis. Nevertheless, the information on candidate genes related to the sepsis in liver has limited.

Understanding protein physiology and function requires quantitative analysis of proteins. More importantly, proteomic studies have been widely applicated to identify various pathways, functions, and biomarkers related to the disease. To date, blood is the primary research object in most proteomic studies of sepsis, and organ proteomics is little understood. It has been shown previously that the liver is one of the most vulnerable organs in sepsis due to its metabolism and coagulation. Thus, as the research object for sepsis proteomics, the liver should play an important role in the identification of liver injury markers. In this regard, we performed quantitative proteomics using the Tandem Mass Tages (TMT)-based methods to explore the key features of proteins or pathways involved in liver injury model of septic rats.

## Materials and Methods

### Ethics statement

All experiments and procedures in this study were performed in strict accordance with protocols approved by the Institutional Animal Care and Use Committee of Shenzhen People’s Hospital Laboratory Animal Center (approval number: AUP-220516-TDE-0341-01).

### Sepsis mouse models

A total of 12 healthy Sprague–Dawley (SD) male rats (180–200 g) aged from 6 to 8 weeks were adaptively housed in individual cages in the SPF (specific pathogen free) animal room at a room temperature of 20–26 °C, alternating day and night, every 12 h, and given free access to water. These SD rats were from Zhuhai Bestest Biotechnology Co., Ltd, License No.: SCXK (Guangdong, China) 2020-0051. All animals were provided with a standard autoclaved commercial diet and filtered water. Our research model was built after 7 days of adapting rats’ diets. These lab rats were grouped into two groups: treatment (*N* = 9) and control (*N* = 3) groups; only three mice from the treatment group survived and were used for subsequent experiments. For the treatment group, the rats were intraperitoneally injected with LPS (*Escherichia coli* O55:B5 containing LPS; #L2880; Sigma-Aldrich, St. Louis, MO, USA). These rats were given an injection volume of 8 mg/kg/day, once injected. A total of 8 h later, these individuals received subcutaneous rehydration with saline at a volume of 60 mL/kg/day. For the control group, the rats received an equal volume of normal saline. To determine whether the sepsis model was successful, we observed whether the rats had symptoms of sepsis. At 6 h after receiving LPS injection, we observed that the rats in the treatment group showed symptoms of sepsis, including the erect hair, lower skin temperatures, and unresponsiveness. After the experiment, the intraperitoneal injection of sodium pentobarbital (150–200 mg/kg) was used for the rat euthanasia.

### Sample and protein preparation

The sampling was performed after the sepsis model construction at 24 h. The liver tissue samples for three treatments and three control groups were collected and stored in the centrifuge tubes after washing with sterile ddH_2_O and frozen in liquid nitrogen until use. Briefly, the preparation of the working liquid was accomplished by thoroughly mixing RIPA lysate with a protease inhibitor and cooling it on ice. Samples with 1,000 μL working liquid were fully dissolved on ice. Centrifugation was conducted under 14,000 rpm/min at 4 °C for 15 min. The supernatant was then transferred to new centrifuge tubes. BCA Quantification kit (P0009; Beyotime Biotechnology, Haimen, Jiangsu, China) was used to detect the protein concentration for each sample following the manufacturer’s instructions.

### TMT labeling

After digestion with trypsin, the resulting peptide for each sample was redissolved and then labeled using the TMT Isobaric Label Reagent Set (90110; Thermo Fisher Scientific, Waltham, MA, USA) following the instruction of manufacturers. For the sodium deoxycholate (SDC) cleaning, 2% trifuoroacetic acid (TFA) was applied after TMT labeling. After that, the C18 column was used to desalt the TMT-labeled peptides with vacuum centrifugation.

### HPLC fractionation

TMT-labeled peptides were separated by the Column (150 mm × 2.1 mm, 2.5 μm; XBridge BEH C18 XP; Waters Corporision, Milford, MA, USA). The mobile phases were 10 mM ammonium acetate aqueous solution (Solvent A) and Acetonitrile (ACN)/water (90:10, v/v, ammonium acetate 10 mM) (Solvent B), pH was adjusted to 10 with ammonia water. Procedures of solvent gradient elution were as follows: 2 min, 95% A, 5% B; 40 min, 70–95% A, 5–30% B; 40 min, 60–70% A, 30–40% B; 4 min: 10–60% A, 40–90% B; 2 min: 10% A, 90% B; 2 min: 2% A, 98% B. After fractionating the peptides into 60 fractions (1 min intervals), 12 fractions were then pooled. Before LC-MS/MS analysis, samples were vacuum-dried and stored at −80 °C.

### LC-MS/MS analysis

In total, 2 μg peptides for each sample were separated by the EASY-nLC1200 HPLC system (Thermo Fisher Scientific, Waltham, MA, USA) interfaced with the Q Exactive HFX Orbitrap instrument (Thermo Fisher Scientific, Waltham, MA, USA). The mobile phases were water with 0.1% FA (A) and 99.9% ACN with 0.1% FA (B). Chromatographic separation was performed using a reversed-phase C18 column (100 μm ID × 15 cm, 1.9 μm; Reprosil-Pur 120 C18-AQ; Dr. Maisch, Ammerbuch, Baden-Wuerttemberg, Germany). Peptides were eluted with a 90 min linear gradient at 300 nL/min flow rate: 2–5% solvent B, 2 min; 5–22% solvent B, 68 min; 22–45% solvent B, 16 min; 45–95% solvent B, 2 min, and 95% solvent B, 2 min. Data-dependent mode (DDA) was employed for mass spectrometry. The MS1 full scan was from 350–1,600 m/z, and data were obtained at a high resolution of 120,000 (200 m/z). For MS2, the resolution was set to 45k (110 m/z). In addition, peaks with charges greater than 6 were excluded from the DDA procedure due to the dynamic exclusion time window of 45 s.

### Proteomics data analysis

The raw data for LC-MS/MS were processed using the Sequest HT search engine built into Proteome Discoverer (PD) ver. 2.4.0.305 software. An analysis of MS spectra lists was performed against the protein sequence database (UniProt-Rattus norvegicus-10116-2021-8.fasta), with the Carbamidomethyl (C), TMT 6 plex (K), and TMT 6 plex (N-term) as a fixed modification and Oxidation (M) and Acetyl (Protein N-term) as variable modifications. The parameters were set as follows: specific enzyme was trypsin; maximum missed cleavage number was two; peptide tolerance was 10 ppm; MS/MS tolerance 0.02 Da; FDR ≤ 0.01, only quantitated unmodified unique peptide. Unique peptide and Razor peptide were used for protein quantification and total peptide amount for normalization. All the other parameters were reserved as default.

Differentially expressed proteins (DEPs) were selected as following parameters: unique peptides ≥2 with average ratio-fold change >2 (upregulation) or <0.5 (downregulation), as well as *P*-value < 0.05. Hierarchical clustering was utilized for analyzing the DEPs. The ClusterProfile (Wu et al., 2021) in the R package was carried out to investigate the Gene Ontology (GO) and Kyoto Encyclopedia of Genes and Genomes (KEGG) pathways of DEPs. The STRING (ver11.0) database was carried out to build protein-protein interaction (PPI) networks among the DEPs.

### Parallel reaction monitoring (PRM) for verification

The PRM method was used to verify the reliability of results obtained from TMT analysis, 18 DEPs were selected for quantification by PRM, Peptides used in PRM were prepared as described for TMT. The same amount of iRT standard peptide (Thermo Fisher Scientific, Waltham, MA, USA) was added to each sample for adjusting retention time and LC–MS quality control. For each sample, 2 μg of total peptides were separated and analyzed using nano-UPLC (EASYnLC1200) coupled to a Q Exactive HFX Orbitrap instrument (Thermo Fisher Scientific, Waltham, MA, USA) with a reversed-phase column (100 μm ID × 15 cm, 1.9 μm; Reprosil-Pur 120 C18-AQ; Dr. Maisch, Ammerbuch, Baden-Wuerttemberg, Germany). The separation of samples was performed by using a 90 min gradient of phase A (0.1% FA in water and 2% ACN) and phase B (0.1% FA, 80% ACN) at 300 nL/min flow rate. The parameter of “chromatography in the DDA mode” was also selected for the PRM mode test. The target peptide lists selected from the DDA results were imported into the Inclusion list of the Xcalibur PRM method editing module by Skyline software. PRM was conducted with Orbitrap analyzer at a resolution of 15,000 (@200 m/z) in centroid and positive modes to hit an automatic gain control (AGC) target of 1 × 10^5^. Inclusion ions were fragmented by high-energy collisional dissociation (HCD) with an isolation window of 0.7 m/z and a normalized collision energy (NCE) of 27%. Three replicates for each sample were employed.

For data analysis, all of the PRM-MS data were processed with Skyline. Five most intense product ions were picked for quantification. The peptide intensity was determined by the sum of the product ions intensity and the quantified protein intensities was determined by the median intensity of peptides which belong to the same protein. Then the ratio between control and test was calculated by the mean protein intensity in different groups.

### Statistical analysis

All data were presented as mean ± standard deviation (SD). Statistical analysis was performed by GraphPad Prism version 9.0 software (GraphPad, San Diego, CA, USA). Results were compared with the *t*-test or one-way analysis of variance (ANOVA). Statistical significance was defined as a *P*-value < 0.05.

## Results

### Proteomic data statistics

**Figure 1:**
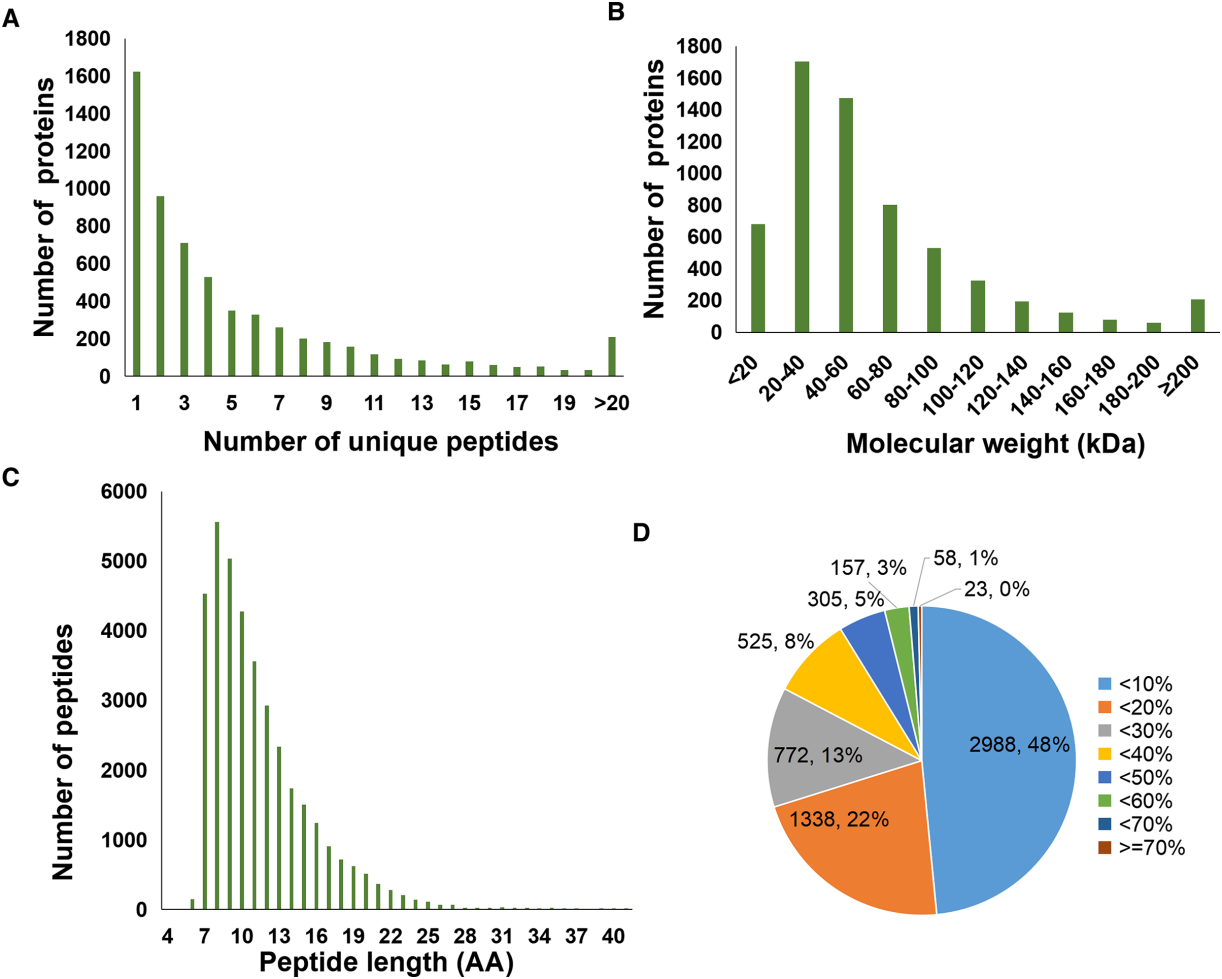
Statistics information of proteomic data in all samples. (A) Number distribution of unique peptides. (B) Molecular weight distribution of proteins. (C) Number distribution of peptide length. (D) Coverage distribution of identified proteins.

A total of 37,012 peptides were identified from six samples. Here, Fig. 1A showed the distribution of unique peptides. The majority of peptides ranged from 7 to 10 amino acid lengths were observed (Fig. 1C). In addition, a total of 6,166 proteins was identified in the present study, and the molecular weight distribution of them was shown in Fig. 1B. Further, we found that a total of 2,988 identified proteins (48% of the total) had a lower coverage, with a value of <10% (Fig. 1D).

### Analysis of differentially abundant proteins between sepsis model and control

**Figure 2:**
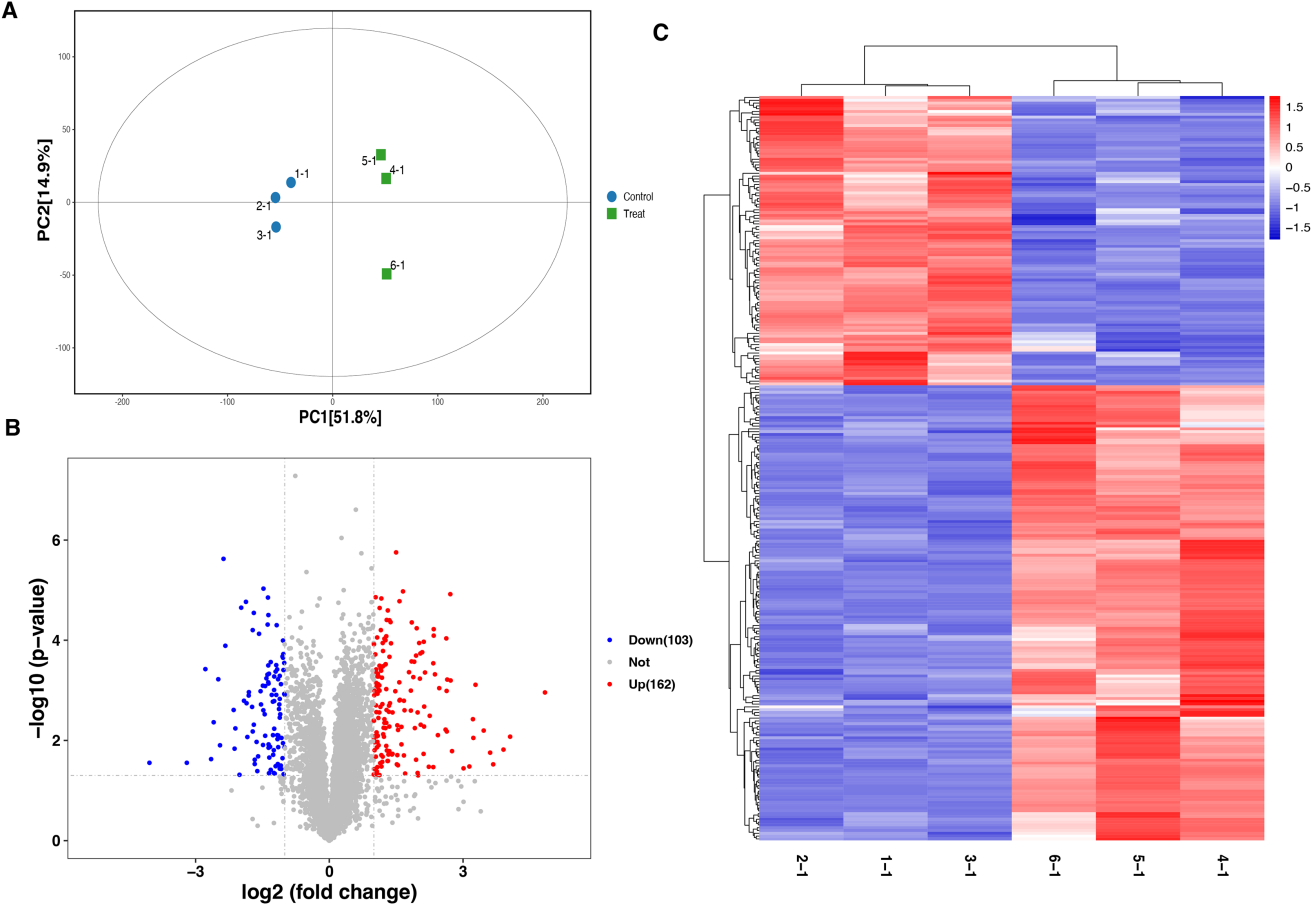
Analysis of DEPs between sepsis model and control. (A) PCA analysis of all samples. (B) Volcano plots display the DEPs between of sepsis rat and control. (C) Heatmap plots indicate the DEPs between sepsis rat and control.

A total of 6,166 proteins were identified using the TMT proteomic sequencing in the present study, 5,701 of which were quantitative. The PCA analysis showed that all the samples clustered into two groups, indicating that both the treatment and control groups exhibit good repeatability (Fig. 2A). Compared with the healthy control group, a total of 265 differentially expressed proteins (DEPs) were identified in the septic rat group, including 162 upregulated and 103 downregulated proteins, based on the threshold of Fold change <0.5 or Fold change >2, and *P*-value < 0.05 (Fig. 2B, Table S1). The hierarchical clustering analysis of DEPs is shown in Fig. 2C.

### Functional characterization and enrichment of DEPs

**Figure 3:**
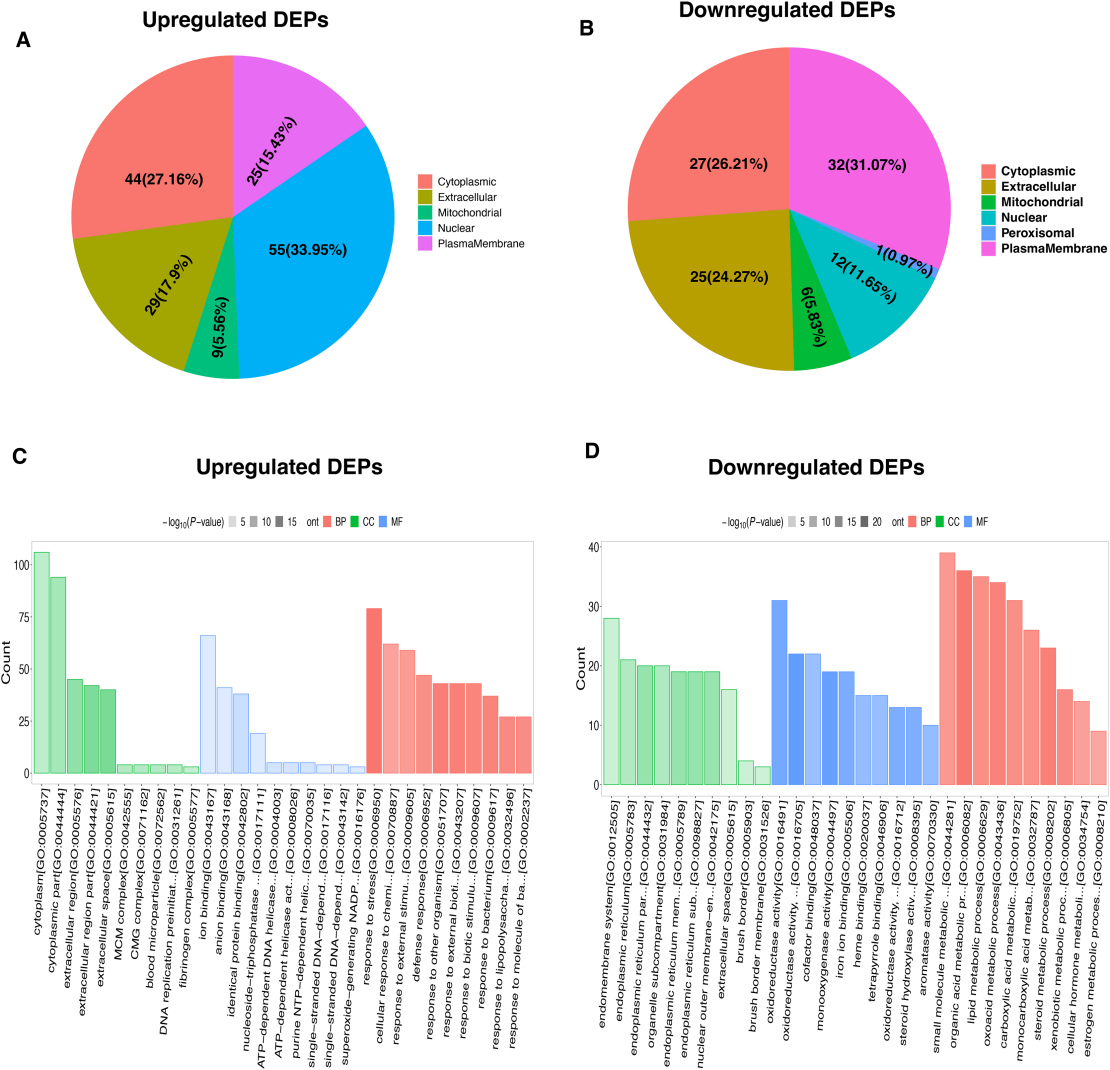
Functional characterization and enrichment of DEPs. Subcellular location of upregulated (A) and downregulated (B) DEPs, respectively. GO enrichment analysis of upregulated (C) and downregulated (D) DEPs.

To characterize the functional features of DEPs, we performed the analysis of subcellular and location and GO annotation for upregulated and downregulated DEPs. The results showed that most of upregulated DEPs were from the nucleus (*n* = 55, 33.95%), followed by the cytoplasm (*n* = 44, 27.16%), extracellular (*n* = 29, 17.9%), plasma membrane (*n* = 25, 15.43%), and Mitochondrial (*n* = 9, 5.56%) (Fig. 3A). In contrast, most of downregulated DEPs were plasma membrane (*n* = 32, 31.07%), followed by cytoplasm (*n* = 27, 26.21%), extracellular (*n* = 25, 24.27%), nucleus (*n* = 12, 11.65%), Mitochondrial (*n* = 6, 5.83%), and peroxisomal (*n* = 1, 0.97%) (Fig. 3B).

Further, the upregulated DEPs divided into three classifications according to the percentage by GO: cellular component (20; 6.92% of the total), molecular function (18; 6.23% of the total), and biological process (251; 86.85% of the total) (Table S2). Figure 3C revealed the top10 GO enrichment results of upregulated DEPs. We observed that the most significant term of CC, BP, and MF was cytoplasm, response to stress, and ion binding, respectively. For the downregulated DEPs, they were classed into 78 BP (77.23% of the total), 17 MF (16.83% of the total), and 6 CC (5.94% of the total) (Table S3). The top10 GO enrichment results of downregulated DEPs was shown in Fig. 3D. The most significant term for CC, BP, and MF was endomembrane system, small molecule metabolic process, and oxidoreductase activity, respectively.

**Figure 4:**
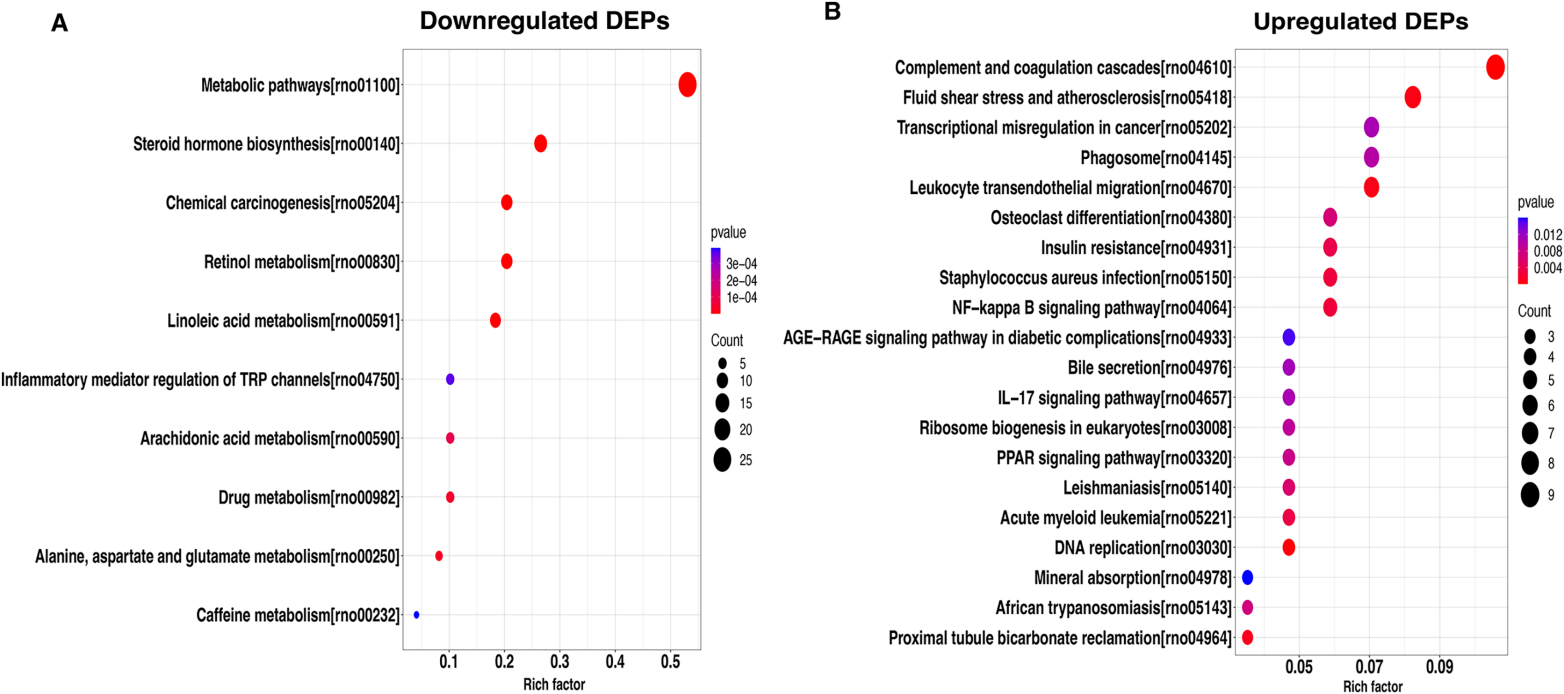
KEGG pathway-based enrichment analysis of downregulated (A) and upregulated (B) DEPs. The size of the circle indicates the enrichment degree of DEPs in the pathway, and the color of the circle indicates the *P*-value of significant enrichment.

To better understand the potential function of these DEPs, we performed a KEGG analysis of upregulated and downregulated DEPs. For down-regulated DEPs, the most significant pathway was the metabolic pathway, followed by steroid hormone biosynthesis and chemical carcinogenesis (Fig. 4A). In contrast, the complement and coagulation cascades, fluid shear stress and atherosclerosis, and transcriptional misregulated in cancer were found to be the top3 most significant pathways for the up-regulated DEPs (Fig. 4B).

### Protein interaction network for DEPs

**Figure 5:**
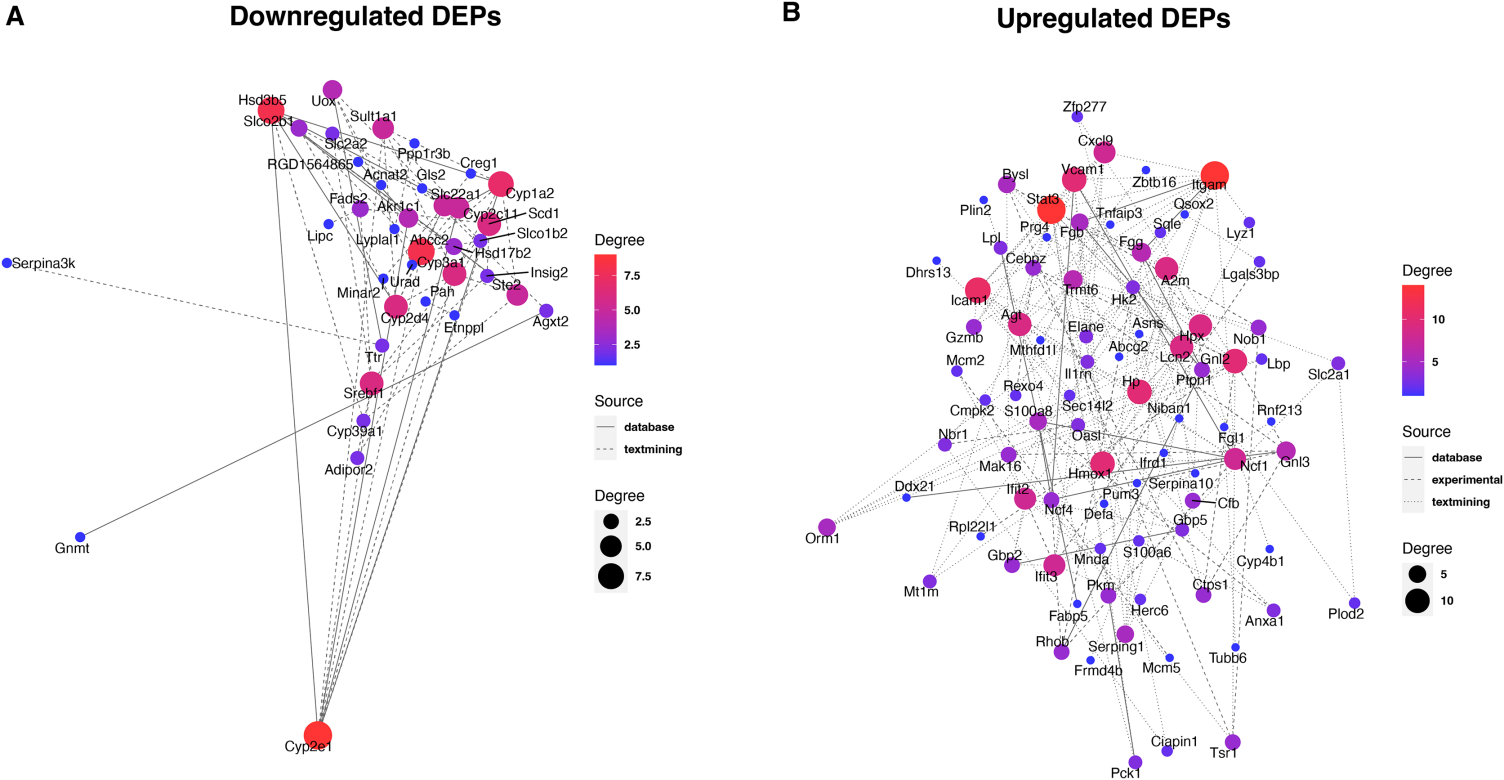
Protein-protein interaction analysis of downregulated (A) and upregulated (B) DEPs.

To reveal the interaction between proteins, we analyzed the PPI network relationships of the upregulated and downregulated DEPs. For the downregulated DEPs, a total of 37 DEPs were connected in the network (Fig. 5A). We found that these DEPs including Cyp2e1, Hsd3b5, Abcc2, and Cyp2d4 had a high linkage degree (degree value > 7) in the network. KEGG enrichment analysis showed that 14 DEPs were significantly enriched into the metabolic pathways (Table 1), two (Cyp2e1 and Hsd3b5) of which had higher degree values in the PPI network.

**Table 1:** Key downregulated and upregulated DEPs based on PPI analysis.

Type	Genes	Term	*P*-value	*q*-value
Down_ regulated	Fads2, Lipc, Uox, Gnmt, Scd1, Cyp1a2, Cyp2e1, Etnppl, Hsd3b5, Urad, Pah, Hsd17b2, Agxt2, Gls2	Metabolic pathways	5.29E−12	2.96E−10
	Hsd3b5, Hsd17b2, Cyp1a2, Cyp2e1	Steroid hormone biosynthesis	1.14E−06	3.18E−05
Up_ regulated	Cfb, Serping1, Itgam, Fgg, A2m, Fgb	Complement and coagulation cascades	4.95E−08	6.24E−06
	Agt, Ptpn1, Pck1, Stat3, Slc2a1	Insulin resistance	4.59E−06	0.000177902
	Hk2, Hmox1, Hpx, Stat3, Slc2a1	HIF-1 signaling pathway	5.42E−06	0.000177902
	Itgam, Ncf1, Vcam1, Icam1, Ncf4	Leukocyte transendothelial migration	5.65E−06	0.000177902
	Plin2, Lpl, Pck1, Fabp5	PPAR signaling pathway	4.18E−05	0.001052557
	Fgg, Cfb, Itgam, Icam1	*Staphylococcus aureus* infection	6.04E−05	0.001268614
	Agt, Stat3, Vcam1, Icam1	AGE-RAGE signaling pathway in diabetic complications	7.31E−05	0.001315105
	Lbp, Tnfaip3, Vcam1, Icam1	NF-kappa B signaling pathway	9.72E−05	0.001530393
	Ncf1, Hmox1, Vcam1, Icam1	Fluid shear stress and atherosclerosis	0.000266984	0.003737778
	Tubb6, Ncf1, Itgam, Ncf4	Phagosome	0.000636007	0.005213993
	Elane, Itgam, Gzmb, Zbtb16	Transcriptional misregulation in cancer	0.000662094	0.005213993

For the upregulated DEPs, a total of 83 DEPs clustered into one PPI network (Fig. 5B). We observed that these DEPs including Itgam, Stat3, Icam1, Gnl2, Hmox1, Vcam1, and Hp had a high linkage degree (degree value > 10) in the network. These results suggested that these DEPs might play a vital role in their network. KEGG enrichment analysis revealed that six DEPs were significantly enriched into the Complement and coagulation cascades pathway (Table 1), of which Itgam had a higher degree value in the PPI network.

**Figure 6:**
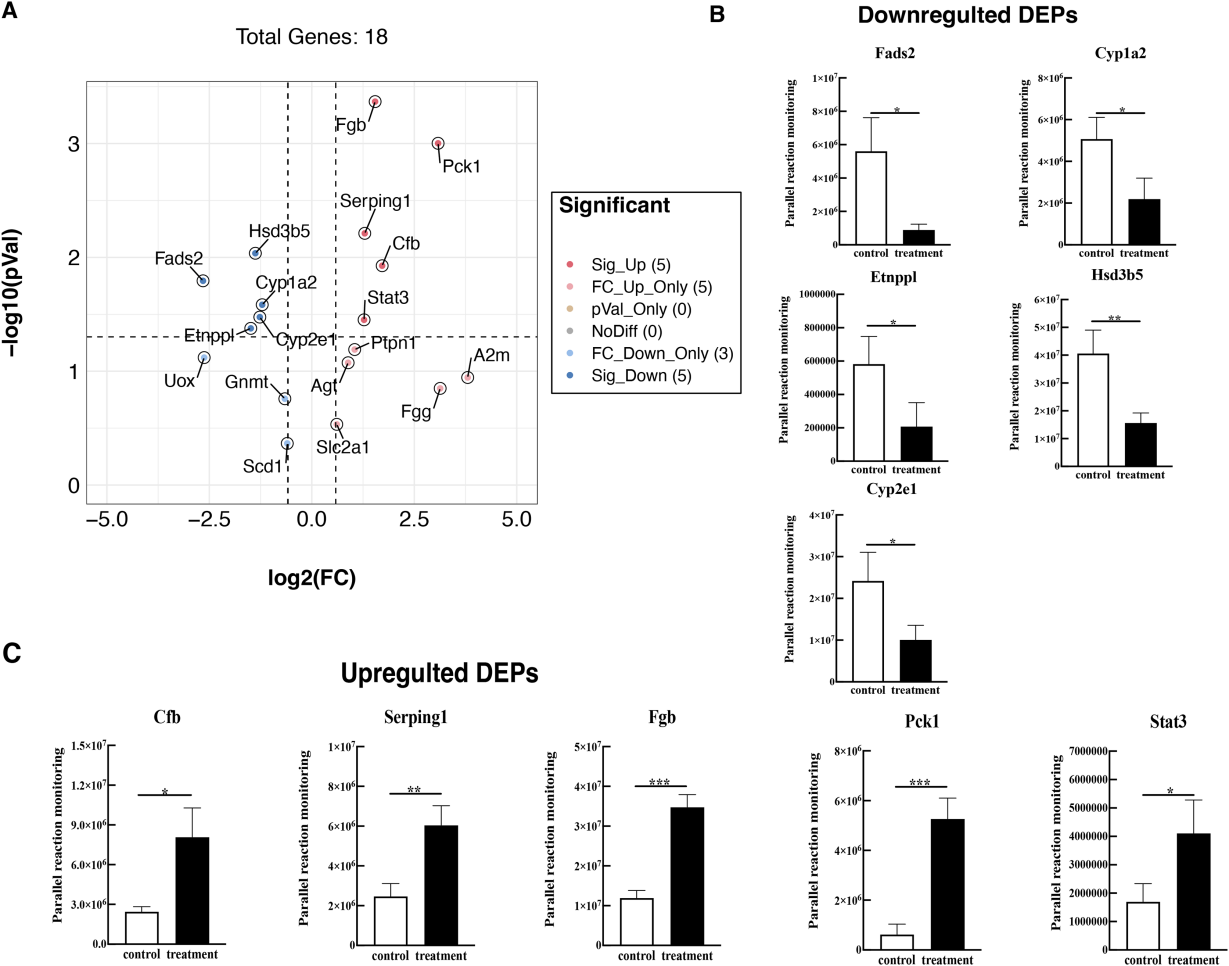
Verified the DEPs by parallel reaction monitoring (PRM). (A) Volcano plots display the difference information of 18 proteins between treatment and control groups. (B) PRM reveals the reliability of downregulated DEPs. (C) PRM reveals the reliability of upregulated DEPs. An asterisk (*) indicates significant difference (*P* < 0.05). Two asterisks (**) indicate significant difference (*P* < 0.01). Three asterisks (***) indicate significant difference (*P* < 0.001).

To further validate these results, 18 proteins were selected for quantification by PRM, including Fads2, Fgb, Cypla2, Cyp2e1, Cfb, Serping1, Fgg, Etnppl, Agt, Hsd3b5, Gnmt, A2m, Pck1, Stat3, Ptpn1, Scd1, Slc2a1, and Uox. The PRM results were consistent with the previous data of the TMT test, 18 promising proteins showed a similar up-regulation or down-regulation both in the two approaches (Fig. 6A, Table S4). Partial results (*P* < 0.05) are shown in Figs. 6B and 6C, supporting the proteomic data.

## Discussion

In the present study, 265 DEPs were detected in the liver of the septic rat model using TMT-based proteomic analyses. These upregulated DEPs were mainly located in the nucleus, whereas most downregulated DEPs were located in the plasma membrane. GO analysis revealed that upregulated DEPs were mainly involved in ion binding, while downregulated DEPs were mainly involved in oxidoreductase activity. Evidence showed that ion channel function might play a vital role in infection and sepsis (D’Elia & Weinrauch, 2018). This means that most of the upregulated DEPs might contribute to the modulation of ion binding by sepsis. In addition, sepsis is known to be closely associated with oxidase activity (Luchtemberg et al., 2008; Kumar et al., 2018; Lopes-Pires, Frade-Guanaes & Quinlan, 2021). This suggested that most of the downregulated DEPs might involve in the regulation of reactive oxygen species and oxidative stress in the body.

Further, KEGG analysis reveals that most down-regulated DEPs were significantly enriched in the metabolic pathway, followed by steroid hormone biosynthesis and chemical carcinogenesis. Evidence has demonstrated that sepsis could lead to sepsis-induced dysfunction and mitochondrial damage, which is suggested as a major cause of cell metabolism disorders in these patients (Wasyluk & Zwolak, 2021). Likewise, Téblick et al. (2022) also highlighted sepsis contributed to the development of severe metabolic alterations. Therefore, we have reason to believe that the DEPs are significantly (*P* < 0.05) enriched into metabolic pathways consistent with previous findings. In other words, these DEPs located in metabolic pathways play an important role in septic liver injury. Moreover, Angstwurm et al. (2003) reported that steroid hormone synthesis is impaired in patients with severe sepsis, suggesting that the steroid hormone synthesis pathway might play a vital role in sepsis development. In addition, a growing body of studies has demonstrated that steroid hormone synthesis was associated with innate immune function (Regan et al., 2013; Patt et al., 2018; Chakraborty, Pramanik & Mahata, 2021). For the upregulated DEPs, we found that most of them were significantly enriched for the complement and coagulation cascades, fluid shear stress and atherosclerosis, and transcriptional misregulated in cancer. Several studies have demonstrated the tight interconnection between the coagulation and complement systems, emphasizing that uncontrolled activation of these enzymatic cascades may negatively impact organ function and result in death during sepsis (Markiewski, DeAngelis & Lambris, 2008; Lupu et al., 2014; Mannes et al., 2021). These results suggested that the pathways might play vital roles in the pathogenesis of septic liver injury.

Identification of key proteins is crucial to understanding the pathogenesis of septic liver injury and developing new diagnostic markers. In the present study, we observed that a total of 37 downregulated DEPs were connected in the network (Fig. 5A), and several DEPs including Cyp2e1, Hsd3b5, Abcc2, and Cyp2d4 had a high linkage degree (degree value > 7) in the PPI network. Notably, Cyp2e1 and Hsd3b5 were both found within the Metabolic pathways. Cyp2e1 is reported to generate reactive oxygen species (ROS) and reactive nitric oxide (RNS) that contribute to cell dysfunction during sepsis (Biswal & Remick, 2007; Abdelmegeed et al., 2017). Hsd3b5 is known to be involved in the production of all classes of steroid hormones that play an essential role in innate immune function (Cutolo et al., 2004; Wang et al., 2020; Xiao et al., 2020). In addition, Abcc2 is reported to be associated with sepsis (Devgun et al., 2012). These results suggested that the key DEPs might involve in the pathogenesis of septic liver injury.

Moreover, we found that a total of 83 upregulated DEPs clustered into one PPI network (Fig. 5B), and these DEPs including Itgam, Stat3, Icam1, Gnl2, Hmox1, Vcam1, and Hp had a high linkage degree (degree value > 10) in the PPI network. KEGG enrichment analysis revealed that six DEPs were significantly enriched into the Complement and coagulation cascades pathway, of which Itgam had a higher degree value in the PPI network. Bu et al. (2020) demonstrated that Itgam was one of key candidate genes in the neonatal sepsis, providing useful information for identifying novel therapeutic markers for neonatal sepsis. Gnl2 was reported to be a potential gene associated with sepsis using the microarray analysis (Li et al., 2018). A previous study reported that STAT3 plays a vital regulatory role in the inflammatory response during sepsis (Williamson et al., 2019). Both Vcam1 (Whalen et al., 2000) and ICAM-1 (Hildebrand et al., 2005) were found to play an important role in the response to polymicrobial sepsis. HMOX1 is reported to be the enzyme responsible for heme scavenging in sepsis (Batra et al., 2020). In addition, Hp is reported to be an important protein involved in the diagnosis of sepsis (Philip, 2012). These results further suggested that these identified DEPs might have important roles in the pathogenesis of septic liver injury. Compared to conventional methods, these markers detected in the liver tissues by proteomics could more directly reflect liver injury with higher specificity. Additionally, they could reveal new pathways to provide a more comprehensive molecular landscape of septic liver injury.

There was a limitation in the present study, with a small sample size. The functions of the identified key candidate genes require confirmation by laboratory data. Although our PRM analysis demonstrated the reliability of predicted results, future investigations aim to confirm the interactions of DEPs underlying the pathogenesis of sepsis.

## Conclusions

In summary, the liver proteomic analyses revealed differentially expressed proteins between the septic rat model and healthy controls. Some key pathways and DEPs have been confirmed to play vital roles in the host immune response. Notably, these DEPs including Fads2, Fgb, Cypla2, Cyp2e1, Cfb, Serping1, Fgg, Etnppl, Agt, Hsd3b5, Gnmt, A2m, Pck1, Stat3, Ptpn1, Scd1, Slc2a1, and Uox were key genes in liver sepsis, which maybe associated with inflammation in sepsis. These findings may further help reveal the pathogenesis of sepsis and serve as potential treatment targets for septic liver injury.

## Supplemental Information

10.7717/peerj.15294/supp-1 Supplemental Information 1 The identified information of differentially expressed significant proteins by TMT.

10.7717/peerj.15294/supp-2 Supplemental Information 2 GO enrichment analysis of upregulated DEPs.

10.7717/peerj.15294/supp-3 Supplemental Information 3 GO enrichment analysis of downregulated DEPs.

10.7717/peerj.15294/supp-4 Supplemental Information 4 Comparision of targeted protein expression according to TMT and PRM approaches.

10.7717/peerj.15294/supp-5 Supplemental Information 5 Author_Checklist_-_Full.

## Additional Information and Declarations

### Data Availability

The following information was supplied regarding data availability: The mass spectrometry proteomics data are available at the ProteomeXchange Consortium *via* the iProX partner repository: PXD037868. The raw measurements are available in the Supplemental Files.
